# CT Findings for Differentiating Pulmonary Mucormycosis From Invasive Pulmonary Aspergillosis, Prior to Invasive Procedure Such as a Biopsy or Surgery: A 22‐Year Single‐Center Experience

**DOI:** 10.1111/myc.70115

**Published:** 2025-09-24

**Authors:** Hyeon Mu Jang, Mi Young Kim, So Yun Lim, Eui‐Jin Chang, Seongman Bae, Jiwon Jung, Min Jae Kim, Yong Pil Chong, Sang‐Ho Choi, Sang‐Oh Lee, Yang Soo Kim, Sung‐Han Kim

**Affiliations:** ^1^ Department of Infectious Diseases, Asan Medical Centre University of Ulsan College of Medicine Seoul Republic of Korea; ^2^ Department of Radiology, Asan Medical Centre University of Ulsan College of Medicine Seoul Republic of Korea

**Keywords:** aspergillosis, CT imaging, mucormycosis

## Abstract

**Objectives:**

Computed tomography (CT) plays a critical role in the early detection and diagnosis of pulmonary invasive mould infection. This study aimed to compare the CT findings of proven invasive pulmonary aspergillosis (IPA) and proven pulmonary mucormycosis (PM) and develop a clinical scoring system based on CT features to differentiate PM from IPA.

**Methods:**

The medical records of the pathology database among adult patients (aged ≥ 18 years) diagnosed with proven IPA or PM between January 2003 and June 2024 were retrospectively reviewed, according to the 2020 European Organisation for Research and Treatment of Cancer criteria. CT scans were reviewed by an experienced radiologist. The primary outcome was CT findings in PM and IPA. We investigated and compared the thoracic CT findings between PM and IPA to identify the predictors of PM compared to IPA prior to invasive diagnostic procedures.

**Results:**

A total of 94 patients were included (60 with IPA and 34 with PM). The most common underlying conditions were malignancy (53.2%) and transplantation (47.9%). In univariable analysis, CT features significantly associated with PM, compared to IPA (*p* < 0.05), included representative lesion size ≥ 4 cm (odds ratio [OR] 3.61, 95% CI 1.48–8.79), consolidation (OR 5.56, 95% CI 1.52–20.38), halo sign (OR 3.33, 95% CI 1.39–8.02), reverse halo sign (RHS) (OR 6.73, 95% CI 2.39–18.98) and airway‐invasive lesion (OR 0.32, 95% CI 0.13–0.78). In multivariate analysis, representative lesion size ≥ 4 cm, RHS, and airway‐invasive lesion were identified as independent predictors of PM, compared to IPA. These three factors were incorporated into a point‐based scoring system (representative lesion size ≥ 4 cm = 11 points; RHS = score 17 points; airway‐invasive lesion = −12 points). A total score of > 8 differentiated PM from IPA with 70.6% sensitivity and 78.3% specificity.

**Conclusions:**

CT findings of large consolidative lesions, the presence of a reverse halo sign, and the absence of airway invasion may aid in the early differentiation of PM from IPA.

## 1 Introduction

Pulmonary invasive mould infection (PIMI) is rare, difficult to diagnose, and associated with high mortality [1, 2]. Computed tomography (CT) plays a critical role in diagnosing PIMI, including invasive pulmonary aspergillosis (IPA) and pulmonary mucormycosis (PM) [1, 3, 4, 5, 6]. Differentiating PM from IPA is particularly important, as voriconazole (the standard treatment for aspergillosis) is ineffective against mucormycosis. However, definitive diagnosis of invasive mould infections, especially PM, remains challenging. Currently, no antigen testing exists for mucormycosis, and fungal culture has low sensitivity for detecting Mucorales species [7]. Some CT findings have been reported to help distinguish PM from IPA [3, 4, 5, 6]. Nevertheless, prior studies are limited by small sample sizes [4, 5] and inconsistent diagnostic certainty between IPA and PM, making comparisons difficult. In particular, many studies compared probable IPA cases with proven PM cases, introducing potential bias [6]. To address these limitations, we analysed more than 20 years of biopsy‐proven cases of IPA and PM, comparing CT findings and developing a clinical scoring system based on imaging features to differentiate PM from IPA, prior to invasive procedures such as a biopsy or surgery.

## 2 Methods

### 2.1 Study Population

This study was conducted at Asan Medical Centre, a 2700‐bed tertiary‐care teaching hospital in Seoul, South Korea. We retrospectively reviewed the medical records of the pathology database among adult patients (aged ≥ 18 years) between January 2003 and June 2024. Patients with tissue pathology that met the criteria for proven IPA or PM were included. Patients were excluded whose CT imaging was difficult to differentiate between active lung cancer and mould infection lesions, under the radiologist's discretion.

The electronic medical record was used to obtain clinical information, as well as laboratory, microbiology, and radiology results, for each patient encounter. Obtained data are: (1) patient attributes (e.g., demographics, comorbid conditions); (2) microbiology data (e.g., galactomannan assay, sterile and non‐sterile culture); and (3) thoracic CT imaging.

### 2.2 Study Outcome

The primary outcome was CT findings in PM and IPA. We investigated and compared the thoracic CT findings between PM and IPA to identify the predictors of PM compared to IPA, prior to invasive diagnostic procedures.

### 2.3 Definitions

All cases of invasive mould infection were defined according to the 2020 criteria of the European Organisation for Research and Treatment of Cancer (EORTC) [1, 2]. Proven invasive mould infection was defined by histopathologic, cytopathologic, or direct microscopic examination of a specimen obtained by needle aspiration or biopsy, showing hyphae or melanized yeast‐like forms with evidence of associated tissue damage. Morphologic features consistent with aspergillosis and mucormycosis were defined as follows:

– Aspergillosis: narrow (3–6 μm wide), septate hyaline hyphae with dichotomous acute angle (45°) branching.
– Mucormycosis: broad, non‐septate hyphae with right angle branching.

### 2.4 CT Evaluation

CT findings were evaluated by an experienced radiologist (Kim, Miyoung) who was blinded to the patients' clinical characteristics and outcomes (Figures S1–S4). Lesions due to old tuberculosis, underlying diseases, and pneumonia were excluded from the CT evaluation. A glossary of imaging definitions was used to categorise pulmonary lesions [7]. Radiological patterns of IPA were classified into three categories: (a) angio‐invasive pneumonia, (b) airway‐invasive pneumonia, and (c) necrotising pneumonia [7, 8, 9].

Angio‐invasive features included macro‐nodules, mass‐shaped consolidations, infarct‐shaped consolidations, and/or the halo sign (HS). The halo sign was defined as a ground‐glass opacity (GGO) surrounding a nodule or mass. Necrotising pneumonia was indicated by cavitary lesions, air‐crescent signs, the reverse halo sign (RHS), and internal low attenuation compared with muscle. According to the Fleischner Society [10], RHS includes both typical RHS and the bird's nest sign (BNS), as described in our previous study [6]. A typical RHS (more organised, pneumonia‐like appearance) was defined as a mass‐like lesion with central GGO or central lucency surrounded by a ring of consolidation, regardless of size. A BNS (more necrosis‐like appearance) was defined as a mass‐like lesion with central necrotic low attenuation, small cavities, and GGO, surrounded by a ring of consolidations > 3 cm in diameter. Airway‐invasive patterns included clusters of centrilobular nodules (< 1 cm), peribronchial consolidations, peribronchial GGO, small airway lesions, and/or tracheobronchial wall thickening.

The representative lesion on CT in terms of size, dominant location, and tissue plane invasion was also assessed. The representative lesion was defined as the one with the largest and most characteristic appearance. Size was measured as the longest diameter of the mass or consolidation (excluding GGO). Dominant location was classified as central (inner half of the lung near the tracheobronchial tree) or peripheral (outer half of the lung). Tissue plane invasion was defined as the extension of the lesion across anatomical boundaries such as lung fissures, diaphragm, chest wall, or pleura, rather than being contained within a single lobe.

### 2.5 Statistical Analysis

Categorical variables were compared using the *χ* ^2^‐test or Fisher's exact test, as appropriate. Continuous variables were compared using Student's *t*‐test or the Mann–Whitney *U*‐test. Receiver operating characteristic (ROC) analysis was used to determine the optimal cut‐off value for lesion size to differentiate PM from IPA; the selected cut‐off value was rounded to the nearest integer.

Multivariable analysis was performed using logistic regression to identify the predictors of PM compared to IPA. Two models were developed: Model I included CT signs that are simple and clinically accessible for non‐radiologists, while Model II included all CT signs assessed by an experienced radiologist. In the univariable analysis, CT findings predictive of PM at *p* < 0.05 were included in the multivariable model using forward selection. A *p*‐value of < 0.05 was considered statistically significant.

To aid clinical interpretation, a point‐scoring system was developed based on the regression coefficients from Model 1. Each coefficient was multiplied by 10 and rounded to the nearest integer. ROC analysis was used to assess the sensitivity and specificity of the scoring system.

Additional analyses were performed, (1) by symptom duration, (2) among patients who did not receive any antifungal medication, and (3) including host factors, diagnostic testing, and CT findings. Calculations were performed with IBM SPSS Statistics Version 23 for Windows.

## 3 Results

### 3.1 Patient Clinical Characteristics

A total of 99 patients with proven PIMIs (diagnosed by histopathologic examination of sterile tissue) were identified. Of these, five were excluded due to difficulty differentiating between active lung cancer and mould infection lesions. The remaining 94 patients were included in the final analysis, comprising 60 cases of IPA (63.8%) and 34 cases of PM (36.2%) during the study period. The baseline clinical characteristics are summarised in Table 1. The median age was 58.5 (IQR 52–65), and 67.0% were male. The most common underlying conditions were malignancy (53.2%), transplant (47.9%), cardiac disease (38.3%), and chronic lung disease (34.0%). Compared with PM, IA was more frequently associated with solid organ transplant (17.6% [6/34] vs. 40.0% [24/60]; *p* = 0.02) and chronic lung disease (14.7% [5/34] vs. 45.0% [27/60]; *p* < 0.01), whereas mucormycosis was more commonly associated with hematologic malignancy (58.8% [20/34] vs. 26.7% [16/60]; *p* < 0.01), and hematologic stem cell transplant (26.5% [9/34] vs. 10.0% [6/60]; *p* = 0.03). IPA was significantly more likely to meet the EORTC criteria for galactomannan assay than PM (50.0% [30/60] vs. 14.7% [5/34]; *p* < 0.01) (Table S1).

**TABLE 1:** Clinical characteristics of proven pulmonary invasive mould infection.

Characteristic	Aspergillosis (*N* = 60)	Mucormycosis (*N* = 34)	*p*
Age (years), median (IQR)	59.5 (53–65)	55.5 (51–64)	0.38
Male sex	40 (66.7)	23 (67.6)	0.92
Malignancy	27 (45.0)	23 (67.6)	0.34
Solid tumour	13 (21.7)	5 (14.7)	0.40
Hematologic malignancy	16 (26.7)	20 (58.8)	< 0.01
Neutropenia < 1500	14 (23.3)	10 (29.4)	0.51
Prolonged neutropenia	8 (13.3)	7 (20.6)	0.35
Transplant	30 (50.0)	15 (44.1)	0.58
Hematologic stem cell transplant	6 (10.0)	9 (26.5)	0.03
Solid organ transplant	24 (40.0)	6 (17.6)	0.02
Chronic lung disease	27 (45.0)	5 (14.7)	< 0.01
Cardiac disease	23 (38.3)	13 (38.2)	0.99
Diabetes mellitus	17 (28.3)	15 (44.1)	0.12
Chronic kidney disease	6 (10.0)	8 (23.5)	0.07
Dialysis	1 (1.7)	1 (2.9)	0.99
Autoimmune disease with immunosuppressants	2 (3.3)	1 (2.9)	0.99

### 3.2 CT Imaging Features of PM and IPA

A comparison of CT findings is presented in Table 2. The median size of the representative lesions (cm; median size [interquartile range {IQR}]) was significantly larger in PM than in IPA (5.0 cm [IQR 3.9–8.0] vs. 3.0 cm [IQR 1.6–5.6], *p* < 0.01). The cut‐off value for the size of the representative lesion for PM probability was calculated using ROC analysis (Figure S5), which was identified as 4 cm for clinical application after rounding to the nearest integer from 3.85 cm. A representative lesion size of ≥ 4 cm was more frequently observed in PM than in IPA (67.6% [23/34] vs. 36.7% [22/60], *p* < 0.01).

**TABLE 2:** Comparison of computed tomography findings between pulmonary mucormycosis (PM) and invasive pulmonary aspergillosis (IPA).

Imaging	Aspergillosis (*N* = 60)	Mucormycosis (*N* = 34)	*p*
Size of representative lesion (target lesion), median (Q1, Q3)	2.95 (1.6–5.55)	4.95 (3.9–8.0)	< 0.01
Size of representative lesion (target lesion)			< 0.01
< 4 cm	38 (63.3)	11 (32.4)	
≥ 4 cm	22 (36.7)	23 (67.6)	
Angioinvasive form (macronodules, consolidation, halo sign)	54 (90.0)	33 (97.1)	0.21
Macronodule	37 (61.7)	21 (61.8)	0.99
Single	10 (16.7)	7 (20.6)	
Multiple	27 (45.0)	14 (41.2)	
Consolidation	39 (65.0)	31 (91.2)	< 0.01
Air bronchogram	17 (28.3)	20 (58.8)	< 0.01
Mass‐shaped consolidation	37 (61.6)	31 (91.2)	< 0.01
Single	27 (45.0)	23 (67.6)	
Multiple	10 (16.7)	8 (23.5)	
Infarct‐shaped consolidation	12 (20.0)	6 (17.6)	0.78
Single	4 (6.7)	0 (0.0)	
Multiple	8 (13.3)	6 (17.6)	
Halo sign	18 (30.0)	20 (58.8)	< 0.01
Necrotizing pneumonia form (reverse halo sign, cavitary lesion ± air‐crescent sign, internal low attenuation)	33 (55.0)	26 (76.5)	0.03
Cavitary lesion	15 (25.0)	7 (20.6)	0.80
Air‐crescent sign	4 (6.7)	5 (14.7)	0.27
Reverse halo sign	7 (11.7)	16 (47.1)	< 0.01
Internal low attenuation	17 (28.3)	19 (55.9)	< 0.01
Airway‐invasive form (clusters of centrilobular nodules, peribronchial consolidation, peribronchial ground‐glass opacity, small airway lesion, tracheobronchial wall thickening)	34 (56.7)	10 (29.4)	0.01
Clusters of centrilobular nodules	28 (46.7)	9 (26.5)	0.05
Peribronchial consolidation	14 (23.3)	3 (8.8)	0.07
Peribronchial Ground‐glass opacity	19 (31.7)	6 (17.6)	0.13
Small airway lesion	9 (15.0)	0 (0.0)	0.02
Tracheobronchial wall thickening	22 (36.7)	8 (23.5)	0.18
Distribution of dominant area			0.04
Central	16 (26.7)	16 (47.1)	
Peripheral	44 (73.3)	18 (52.9)	
Invade tissue plane	9 (15.0)	23 (67.6)	< 0.01
Lung fissures	6 (10.0)	11 (32.4)	
Pleural (visceral and/or parietal)	4 (6.6)	12 (35.3)	

Most cases in both groups showed angio‐invasive features (97.1% [33/34] PM vs. 90.0% [54/60] IPA; *p* = 0.21). However, consolidation (91.2% [31/34] vs. 65.0% [39/60]; *p* < 0.01), air bronchogram (58.8% [20/34] vs. 28.3% [17/60]; *p* < 0.01), and HS (58.8% [20/34] vs. 28.3% [17/60]; *p* < 0.01) were significantly more common in PM. Necrotising pneumonia patterns were also more prevalent in PM (76.5% [26/34] vs. 55.0% [33/60]; *p* = 0.03), particularly the RHS (47.1% [16/34] vs. 11.7% [7/60]; *p* < 0.01). In contrast, airway‐invasive lesions were more frequently observed in IPA (29.4% [10/34] vs. 56.7% [34/60]; *p* = 0.01).

### 3.3 Independent Predictors of PM and a Scoring System for Pulmonary Mucormycosis

The results of logistic regression analyses, using CT findings, for Model I and Model II are shown in Table 3 and Table S2, respectively. In Model I, which included only CT signs that are straightforward for non‐radiologists to evaluate, univariable analysis identified the following predictors of PM, compared to IPA (*p* < 0.05): representative lesion ≥ 4 cm (OR 3.612, 95% CI 1.48–8.79); consolidation (OR 5.564, 95% CI 1.519–20.384); HS (OR 3.333, 95% CI 1.385–8.022); RHS (OR 6.730, 95% CI 2.387–18.979); and airway‐invasive lesion (OR 0.319, 95% CI 0.130–0.781). In the multivariable analysis, the following were independent predictors of PM, compared to IPA: representative lesion size ≥ 4 cm (OR 2.99, 95% CI 1.11–8.05; *p* = 0.03); RHS (OR 5.62, 95% CI 1.85–17.04; *p* < 0.01); and airway‐invasive lesion (OR 0.29, 95% CI 0.10–0.80; *p* < 0.01). In Model II, which included all CT imaging signs evaluated by an experienced radiologist, univariable analysis found the following to be significantly associated with PM, compared to IPA (*p* < 0.05): central dominance (OR 2.444, 95% CI 1.010–5.915), tissue plane invasion (OR 11.848, 95% CI 4.319–32.504), representative lesion size (≥ 4 cm) (OR 3.612, 95% CI 1.483–8.794), consolidation (OR 5.564, 95% CI 1.519–20.384), HS (OR 3.333, 95% CI 1.385–8.022), RHS (OR 6.730, 95% CI 2.387–18.979); and internal low attenuation (OR 3.204, 95% CI 1.330–7.721). In the corresponding multivariable analysis, independent predictors for PM, compared to IPA, were tissue plane invasion (OR 11.652, 95% CI 3.911–34.713) and RHS (OR 6.564, 95% CI 1.953–22.056).

**TABLE 3:** Predictors of pulmonary mucormycosis by univariable and multivariable logistic regression analysis (model I).

Imaging	Univariable		Multivariable^a^	
	Odd ratio (95% CI)	*p*	Odd ratio (95% CI)	*p*
Size ≥ 4 cm	3.612 (1.483–8.794)	0.005	2.99 (1.11–8.05)	0.03
Angioinvasive form				
Macronodule	0.996 (0.419–2.367)	0.993		
Micronodule‐multiple	0.741 (0.232–2.367)	0.613		
Consolidation	5.564 (1.519–20.384)	0.010		
Halo	3.333 (1.385–8.022)	0.007		
Necrotizing pneumonia form				
RHS	6.730 (2.387–18.979)	< 0.001	5.62 (1.85–17.04)	< 0.01
Cavity	0.778 (0.282–2.149)	0.628		
Air crescent	2.414 (0.602–9.683)	0.214		
Airway invasive form	0.319 (0.130–0.781)	0.012	0.29 (0.10–0.80)	0.01

^a^ The forward selection method was used to build model I as a stepwise regression. A *p*‐value of < 0.05 was considered to indicate statistical significance.

Based on the multivariable analysis from Model I, a point‐scoring system was developed (Table 4, Table S3, and Figure S6). The following variables were included: representative lesion size ≥ 4 cm (score = 11), RHS (score = 17), and airway‐invasive lesion (score = −12). A total score of > 8 predicted PM with a sensitivity of 70.6% and a specificity of 78.3%. The area under the receiver operator characteristic curve (AUC) was 0.80 (95% CI 0.75–0.85).

**TABLE 4:** Diagnostic performance of the scoring system for differentiating pulmonary mucormycosis from invasive pulmonary aspergillosis.

Score	PM (*N* = 34)	IPA (*N* = 60)	Sensitivity	Specificity
−13.0	94	0	100 (90–100)	0 (0–6)
−6.5	72	22	94 (80–99)	33 (22–47)
−0.5	59	35	88 (73–97)	52 (38–65)
2.5	39	55	71 (53–85)	75 (62–85)
8.0	37	57	71 (53–85)	78 (66–88)
13.5	21	73	47 (30–65)	92 (82–97)
16.5	14	80	29 (15–47)	93 (84–98)
22.5	9	85	20 (9–38)	97 (88–100)
29.0	0	94	0 (0–10)	100 (94–100)

*Note:* Three independent predictors were used to build the scoring system: the size of representative lesion ≥ 4 cm (a), reverse halo sign (b), and airway invasive lesion (c). The total score is the sum of the three predictors (a = score 11, b = score 17, and c = score −12). The optimal cut‐off is score of 8.0 (sensitivity 71%, specificity 78%).

We performed additional analyses, of which results are shown in Appendix S1 (Tables S4–S7). Of 94, 28 patients did not have any information on their patient notes regarding symptom and/or its duration. There were data regarding symptom and/or its duration for 66 patients. We performed subgroup analyses by symptom duration: (a) ≤ 7 days (1 week), (b) 7–28 days (1–4 weeks), and (c) > 28 days (4 weeks) (Table S4). Among a total of 94 patients, almost two thirds of the patients (64.8% [61/94]) did not receive any empiric fungal medications until CT imaging was checked. Only about 20% (22.3% [21/94]) had received empiric fungal medications for more than 7 days by the time of CT imaging was performed. We performed subgroup analyses on 61 patients who did not receive any antifungal medication (Tables S5 and S6). We also performed additional analyses including host factors, diagnostic testing, and CT findings (Table S7).

## 4 Discussion

Early differentiation of PM from IPA, prior to invasive diagnostic procedures, is critical for selecting appropriate empirical antifungal therapy in immunocompromised patients. To aid in this differentiation, we comprehensively reviewed biopsy‐proven cases of PM and IPA over a 22‐year period, comparing their CT findings. Our analysis identified three key imaging features that help distinguish PM from IPA: (a) a representative lesion size of ≥ 4 cm, (b) the presence of RHS, and (c) the absence of airway‐invasive lesions. These features were incorporated into a novel point‐scoring system to predict PM.

Mucorales are known for their aggressive and destructive behaviour [7, 11, 12, 13, 14]. While the maximum axial diameter of IPA lesions ranges from 1 to 6.9 cm (mean, 2.5 ± 1.9 cm), [15] lesions in PM range from 2.1 to 11.9 cm, with a median of 4.2 cm [16]. To date, no studies have directly compared lesion sizes between IPA and PM. Our previous study [6] also did not assess lesion size. In this study, however, a representative lesion of ≥ 4 cm emerged as an independent predictor of PM compared to IPA. The median size of representative lesions in PM was significantly larger than in IPA (5.0 cm [IQR, 3.9–8.0] vs. 3.0 cm [IQR, 1.6–5.6], *p* < 0.01). In the univariable and multivariable analyses, a lesion size of ≥ 4 cm was associated with PM, with an OR of 3.612 (95% CI, 1.48–8.79) and 2.99 (95% CI, 1.11–8.05), respectively. This association likely reflects the more invasive nature of Mucorales, which frequently involve larger pulmonary vessels and adjacent organs [7, 11, 12, 13, 14]. Thus, lesion size is a useful predictor of PM and may inform empirical antifungal therapy.

Airway‐invasive aspergillosis is a well‐described subtype of IPA [17], whereas airway involvement in mucormycosis has only been reported in isolated case reports [18, 19, 20]. Few studies have directly compared airway‐invasive features between IPA and PM. In our study, airway‐invasive lesions on CT were negatively associated with PM in the univariable (OR 0.319, 95% CI 0.130–0.781) and multivariable analyses (OR 0.29, 95% CI 0.10–0.80), identifying this feature as a significant independent predictor. This finding may assist clinicians in distinguishing PM from IPA and guiding patient management.

Chamilos et al. [4] previously identified ≥ 10 nodules on CT as a significant predictor of PM (OR, 19.8; 95% CI, 1.94–202.29; *p* = 0.012). In contrast, our study found no significant difference in the frequency of multiple micronodules (≥ 2) between IPA and PM (45.0% [27/60] vs. 41.2% [14/34]; *p* = 0.99). Multiple micronodules were not significant predictors in either Model I (OR 0.741, 95% CI 0.232–2.367; *p* = 0.613) or Model II (OR 0.741, 95% CI 0.232–2.367; *p* = 0.613). The cause of this discrepancy is unclear, but further research is needed to clarify the role of nodule number in differentiating PM from IPA.

Aspergillosis (70%–80%) accounts for most invasive mould infections (IMI) [21, 22, 23, 24, 25, 26, 27], followed by mucormycosis (10%–15%) [21, 22, 23, 24, 25, 26, 27], with other moulds comprising only 5%–15%. Given this epidemiology, our scoring system, developed through direct comparison of IPA and PM (excluding other moulds), may provide practical guidance for empiric antifungal selection. The scoring system is based on three easily accessible CT features: (a) representative lesion size of ≥ 4 cm (score = 11), (b) RHS (score = 17), and (c) airway‐invasive lesion (score = −12). This simple, imaging‐based system offers objective support for clinical decision‐making.

This study has several limitations. First, an invasive procedure, such as a transbronchial lung biopsy, was required to diagnose proven PIMI. Some of the immunocompromised patients with cytopenia (particularly thrombocytopenia) might not have undergone such invasive procedures and not have been included in this study (probable or possible PIMI), which might lead to some selection bias. However, the diagnostic level of certainty through comparing proven PM to proven IPA would provide more useful information about CT findings. The result of this study can be helpful, particularly in cases where a biopsy is needed for accurate diagnoses to guide proper antifungal therapy, in cases of negative results of GM and non‐sterile fungal cultures. Second, we could not evaluate some changes in CT protocol during the long study period. Third, this study was aimed at differentiating only the two most common moulds that cause lung lesions. Other etiologies, such as *Fusarium* and *Scedosporium*, were not considered. Therefore, there may be some limitations to generalise this data. Finally, the number of cases of PM was approximately half of those with IPA. This may reflect the clinical practice of the prevalence of biopsy‐proven invasive mould pneumonia. Despite these limitations, the strength of this study is that it evaluated 22‐year data that comprises only biopsy‐proven invasive pulmonary mould infection. This allowed for the exclusion of uncertain cases of probable and possible IPA and PM, which could likely be chronic and saprophytic forms of mould infection rather than the invasive diseases.

In conclusion, the presence of large consolidations (≥ 4 cm), RHS, and the absence of airway‐invasive features on CT imaging are helpful in the early differentiation of PM from IPA.

## Author Contributions

**Hyeon Mu Jang:** conceptualization, data curation, formal analysis, investigation, methodology, supervision, visualization, validation, writing – original draft, writing – review and editing. **Mi Young Kim:** data curation, investigation. **So Yun Lim:** investigation. **Eui‐Jin Chang:** supervision. **Seongman Bae:** supervision. **Jiwon Jung:** supervision. **Min Jae Kim:** supervision. **Yong Pil Chong:** supervision. **Sang‐Ho Choi:** supervision. **Sang‐Oh Lee:** supervision. **Yang Soo Kim:** supervision. **Sung‐Han Kim:** supervision, software, conceptualization, writing – review and editing, project administration, resources, funding acquisition.

## Conflicts of Interest

The authors declare no conflicts of interest.

## Supporting information


**Appendix S1:** myc70115‐sup‐0001‐AppendixS1.docx.

## Data Availability

The data that support the findings of this study are available from the corresponding author upon reasonable request.

## References

[1] J. Peter Donnelly , S. C. Chen , C. A. Kauffman , et al., “Revision and Update of the Consensus Definitions of Invasive Fungal Disease From the European Organization for Research and Treatment of Cancer and the Mycoses Study Group Education and Research Consortium,” Clinical Infectious Diseases 71, no. 6 (2020): 1367–1376, 10.1093/cid/ciz1008. 31802125 PMC7486838

[2] S. Husain , B. D. Alexander , P. Munoz , et al., “Opportunistic Mycelial Fungal Infections in Organ Transplant Recipients: Emerging Importance of Non‐Aspergillus Mycelial Fungi,” Clinical Infectious Diseases 37, no. 2 (2003): 221–229. 12856215 10.1086/375822

[3] B. D. Alexander , F. Lamoth , C. P. Heussel , et al., “Guidance on Imaging for Invasive Pulmonary Aspergillosis and Mucormycosis: From the Imaging Working Group for the Revision and Update of the Consensus Definitions of Fungal Disease From the EORTC/MSGERC,” Clinical Infectious Diseases 72, no. Supplement_2 (2021): S79–S88, 10.1093/cid/ciaa1855. 33709131

[4] G. Chamilos , E. M. Marom , R. E. Lewis , M. S. Lionakis , and D. P. Kontoyiannis , “Predictors of Pulmonary Zygomycosis Versus Invasive Pulmonary Aspergillosis in Patients With Cancer,” Clinical Infectious Diseases 41, no. 1 (2005): 60–66, 10.1086/430710. 15937764

[5] C. Legouge , D. Caillot , M. L. Chrétien , et al., “The Reversed Halo Sign: Pathognomonic Pattern of Pulmonary Mucormycosis in Leukemic Patients With Neutropenia?,” Clinical Infectious Diseases 58, no. 5 (2014): 672–678, 10.1093/cid/cit929. 24352351

[6] J. Jung , M. Y. Kim , H. J. Lee , et al., “Comparison of Computed Tomographic Findings in Pulmonary Mucormycosis and Invasive Pulmonary Aspergillosis,” Clinical Microbiology and Infection 21, no. 7 (2015): 684.e11–684.e18, 10.1016/j.cmi.2015.03.019. 25882362

[7] A. Skiada , I. Pavleas , and M. Drogari‐Apiranthitou , “Epidemiology and Diagnosis of Mucormycosis: An Update,” Journal of Fungi (Basel, Switzerland) 6, no. 4 (2020): 265, 10.3390/jof6040265. 33147877 PMC7711598

[8] D. M. Hansell , A. A. Bankier , H. MacMahon , T. C. McLoud , N. L. Müller , and J. Remy , “Fleischner Society: Glossary of Terms for Thoracic Imaging,” Radiology 246, no. 3 (2008): 697–722, 10.1148/radiol.2462070712. 18195376

[9] P. M. Logan , S. L. Primack , R. R. Miller , and N. L. Müller , “Invasive Aspergillosis of the Airways: Radiographic, CT, and Pathologic Findings,” Radiology 193, no. 2 (1994): 383–388, 10.1148/radiology.193.2.7972747. 7972747

[10] P. M. Logan and N. L. Müller , “High‐Resolution Computed Tomography and Pathologic Findings in Pulmonary Aspergillosis: A Pictorial Essay,” Canadian Association of Radiologists Journal 47, no. 6 (1996): 444–452. 8943916

[11] G. Petrikkos and C. Tsioutis , “Recent Advances in the Pathogenesis of Mucormycoses,” Clinical Therapeutics 40, no. 6 (2018): 894–902, 10.1016/j.clinthera.2018.03.009. 29631910

[12] R. A. Murphy and W. T. Miller, Jr. , “Pulmonary Mucormycosis,” Seminars in Roentgenology 31, no. 1 (1996): 83–87, 10.1016/s0037-198x(96)80043-5. 8838948

[13] R. L. F. Cecil , L. Goldman , and A. I. Schafer , Goldman's Cecil Medicine, Expert Consult Premium Edition—Enhanced Online Features and Print, Single Volume, 24 (Elsevier Health Sciences, 2012). ISBN: 9781437716047.

[14] R. Pfleger , K. Kumar , D. Bell , et al., “Pulmonary Mucormycosis,” Reference article, Radiopaedia.org, accessed January 22, 2025, 10.53347/rID-28658.

[15] S. Sonnet , C. H. Buitrago‐Téllez , M. Tamm , S. Christen , and W. Steinbrich , “Direct Detection of Angioinvasive Pulmonary Aspergillosis in Immunosuppressed Patients: Preliminary Results With High‐Resolution 16‐MDCT Angiography,” AJR. American Journal of Roentgenology 184, no. 3 (2005): 746–751, 10.2214/ajr.184.3.01840746. 15728592

[16] M. M. Hammer , R. Madan , and H. Hatabu , “Pulmonary Mucormycosis: Radiologic Features at Presentation and Over Time,” American Journal of Roentgenology 210, no. 4 (2018): 742–747, 10.2214/AJR.17.18792. 29470162

[17] J. Wu , T. Zhang , J. Pan , et al., “Characteristics of the Computed Tomography Imaging Findings in 72 Patients With Airway‐Invasive Pulmonary Aspergillosis,” Medical Science Monitor 27 (2021): e931162, 10.12659/MSM.931162. 34453030 PMC8409142

[18] J. Mattioni , J. E. Portnoy , J. E. Moore , D. Carlson , and R. T. Sataloff , “Laryngotracheal Mucormycosis: Report of a Case,” Ear, Nose, & Throat Journal 95, no. 1 (2016): 29–39. 26829683

[19] L. C. Luo , D. Y. Cheng , H. Zhu , X. Shu , and W. B. Chen , “Inflammatory Pseudotumoural Endotracheal Mucormycosis With Cartilage Damage,” European Respiratory Review 18, no. 113 (2009): 186–189, 10.1183/09059180.00000709. 20956142

[20] J. R. Schwartz , M. G. Nagle , R. C. Elkins , and J. A. Mohr , “Mucormycosis of the Trachea: An Unusual Cause of Acute Upper Airway Obstruction,” Chest 81, no. 5 (1982): 653–654, 10.1378/chest.81.5.653. 7075293

[21] A. Chakrabarti , H. Kaur , J. Savio , et al., “Epidemiology and Clinical Outcomes of Invasive Mould Infections in Indian Intensive Care Units (FISF Study),” Journal of Critical Care 51 (2019): 64–70, 10.1016/j.jcrc.2019.02.005. 30769292

[22] P. G. Pappas , B. D. Alexander , D. R. Andes , et al., “Invasive Fungal Infections Among Organ Transplant Recipients: Results of the Transplant‐Associated Infection Surveillance Network (TRANSNET),” Clinical Infectious Diseases 50, no. 8 (2010): 1101–1111, 10.1086/651262. 20218876

[23] B. J. Park , P. G. Pappas , K. A. Wannemuehler , et al., “Invasive Non‐Aspergillus Mold Infections in Transplant Recipients, United States, 2001–2006,” Emerging Infectious Diseases 17, no. 10 (2011): 1855–1864, 10.3201/eid1710.110087. 22000355 PMC3311117

[24] H. J. Yoon , H. Y. Choi , Y. K. Kim , Y. J. Song , and M. Ki , “Prevalence of Fungal Infections Using National Health Insurance Data From 2009–2013, South Korea,” Epidemiology and Health 36 (2014): e2014017, 10.4178/epih/e2014017. 25358415 PMC4220602

[25] E. Stavropoulou , A. Huguenin , G. Caruana , et al., “Investigations of an Increased Incidence of Non‐Aspergillus Invasive Mould Infections in an Onco‐Haematology Unit,” Swiss Medical Weekly 154 (2024): 3730, 10.57187/s.3730. 38579310

[26] D. A. Enoch , H. A. Ludlam , and N. M. Brown , “Invasive Fungal Infections: A Review of Epidemiology and Management Options,” Journal of Medical Microbiology 55 (2006): 809–818, 10.1099/jmm.0.46548-0. 16772406

[27] H. I. Shih , Y. T. Huang , and C. J. Wu , “Disease Burden and Demographic Characteristics of Mucormycosis: A Nationwide Population‐Based Study in Taiwan, 2006–2017,” Mycoses 65, no. 11 (2022): 1001–1009, 10.1111/myc.13484. 35713608 PMC9796055

