# Supplementary material for: CT Findings for Differentiating Pulmonary Mucormycosis From Invasive Pulmonary Aspergillosis, Prior to Invasive Procedure Such as a Biopsy or Surgery: A 22‐Year Single‐Center Experience
Source: Mycoses. 2025 Sep 24;68(9):e70115. doi: 10.1111/myc.70115 (PMC12461179; doi:10.1111/myc.70115)
Supplement: Supplementary file 1 — Appendix S1: myc70115‐sup‐0001‐AppendixS1.docx. [file MYC-68-e70115-s001.docx]

**Online Data Supplement**

***CT Scanning Protocol***

Chest CT examinations were performed using 16- or 64-detector equipment; either the SOMATOM Sensation 16 (Siemens Medical Solutions, Forchheim, Germany) or the Lightspeed VCT (General Electric Medical Systems, Milwaukee, WI).

For the 16-detector row scanner, scan parameters were 120 kV and 100 effective mA with dose modulation. Reconstruction intervals were 3-mm thickness and 3-mm interval without gap using the B50 algorithm and 1-mm reconstruction with 5-mm gap using the B60 algorithm.

For the 64-detector row scanner, scan parameters were 120 kV and 100–400 mA with dose modulation. Reconstruction intervals were 3-mm thickness and 3-mm interval without gap using the lung algorithm and 1.25-mm reconstruction with a 5-mm gap using the bone algorithm.

A total of 120 CT scans were obtained with (n=46) and without (n=74) contrast enhancement. All images were viewed at the mediastinal (width, 450 HU; level, 50 HU) and lung window (width, 1500 HU; level, –700 HU) settings of axial images on PACS.

**Supplemental Table 1.** Diagnostic characteristics of the study participants

|  | **Aspergillosis**  **(N=60)** | **Mucormycosis**  **(N=34)** | **P-value** |
| --- | --- | --- | --- |
| Microscopic detection of fungal elements in BAL or bronchial brush indicating Aspergillus | 12 (20.0) | 2 (5.9) | 0.07 |
| Galactomannan (GM) assay  EORTC criteria (+)^1^ | 30 (50.0) | 5 (14.7) | <0.01 |
| Sterile tissue culture (performed)  Aspergillus growth  Mucorales growth  No growth  Not performed | 15 (25.0)  0 (0.0)  15 (25.0)  30 (50.0) | 0 (0.0)  1 (2.9)  26 (76.5)  7 (20.6) | <0.01 |
| Non-sterile culture (performed) Aspergillus growth  Mucorales growth  No growth  Not performed | 25 (41.7)  0 (0.0)  21 (35.0)  14 (23.3) | 2 (5.9)  3 (8.8)  23 (67.7)  6 (17.6) | <0.01 |

^1^One of the following met the EORTC (+) criteria: (1) single serum or plasma: ≥ 1.0; (2) BAL fluid: ≥1.0; (3) single serum or plasma: ≥0.7 and BAL fluid ≥0.8; (4) CSF: ≥1.0

**Supplemental Table 2.** Predictors of pulmonary mucormycosis in univariable analysis (Model II)

| **Imaging** | **Univariable** |  | **Multivariable** |  |
| --- | --- | --- | --- | --- |
|  | Odd ratio  (95% CI) | P-value | Odd ratio  (95% CI) | P-value |
| Dominant (central) | 2.444  (1.010–5.915) | 0.04 |  |  |
| Tissue plane invasion | 11.848  (4.319–32.504) | <0.01 | 11.652  (3.911–34.713) | <0.01 |
| Size ≥3 cm | 6.200  (2.115–18.179) | 0.01 |  |  |
| Size ≥4 cm | 3.612  (1.483–8.794) | <0.01 |  |  |
| **Angioinvasive Form** |  |  |  |  |
| macronodule | 0.996  (0.419–2.367) | 0.99 |  |  |
| Micronodule-multiple | 0.741  (0.232–2.367) | 0.61 |  |  |
| Consolidation | 5.564  (1.519–20.384) | 0.01 |  |  |
| halo | 3.333  (1.385–8.022) | <0.01 |  |  |
| **Necrotizing pneumonia form** |  |  |  |  |
| RHS | 6.730  (2.387–18.979) | <0.01 | 6.564  (1.953–22.056) | <0.01 |
| Cavity | 0.778  (0.282–2.149) | 0.62 |  |  |
| air crescent | 2.414  (0.602–9.683) | 0.21 |  |  |
| Internal low attenuation | 3.204  (1.330–7.721) | <0.01 |  |  |
| **Airway invasive form** |  |  |  |  |
| clusters of centrilobular nodules (<1 cm) | 0.411  (0.165–1.028) | 0.05 |  |  |
| peribronchial Consolidation | 0.318  (0.084–1.199) | 0.09 |  |  |
| peribronchial GGO | 0.462  (0.164–1.303) | 0.14 |  |  |
| small airway lesion | <0.001 | 0.99 |  |  |

^1^The forward selection method was used to build Model II as a stepwise regression. A p-value of <0.05 was considered to indicate statistical significance.

**Supplemental Table 3.** Selected multivariable predictive model for pulmonary mucormycosis and point scoring attributed to each variable.

| **Predictors** | ***B*-coefficient** | **Standard Error** | **Score^1^** |
| --- | --- | --- | --- |
| Size ≥4 cm | 1.095 | 0.505 | 0  11 |
| RHS | 1.727 | 0.565 | 0  17 |
| Airway invasive form on CT imaging | -1.232 | 0.518 | 0  -12 |

^1^The score was calculated by multiplying the coefficient by 10 and rounding to the nearest integer.

**Supplemental Table 4** Comparison of computed tomography findings between pulmonary mucormycosis (PM) and invasive pulmonary aspergillosis (IPA), by symptom duration

| 1. **Symptom duration: ≤ 7 days (N=21)** | | | |
| --- | --- | --- | --- |
| **Imaging** | **Aspergillosis**  (N=15) | **Mucormycosis**  (N=6) | **P-value** |
| Size of representative lesion ≥4 cm | 10 (66.7) | 3 (50.0) | 0.63 |
| Macronodule-multiple | 8 (53.3) | 1 (16.7) | 0.17 |
| Consolidation | 11 (73.3) | 6 (100.0) | 0.2 |
| Halo sign | 7 (46.7) | 5 (83.3) | 0.17 |
| Reverse halo sign | 2 (13.3) | 3 (50.0) | 0.11 |
| Cavity | 1 (6.7) | 2 (33.3) | 0.18 |
| Air crescent | 0 (0.0) | 3 (50.0) | **0.01** |
| Airway-invasive form | 11 (73.3) | 1 (16.7) | **0.04** |
| 1. **Symptom duration: > 7 days and ≤28 days (N=34)** | | | |
| **Imaging** | **Aspergillosis**  (N=17) | **Mucormycosis**  (N=17) | **P-value** |
| Size of representative lesion ≥4 cm | 3 (17.6) | 13 (76.5) | **<0.01** |
| Macronodule-multiple | 9 (52.9) | 7 (41.2) | 0.73 |
| Consolidation | 12 (70.6) | 16 (94.1) | 0.17 |
| Halo sign | 6 (35.3) | 10 (58.8) | 0.30 |
| Reverse halo sign | 1 (5.9) | 9 (52.9) | **<0.01** |
| Cavity | 8 (47.1) | 4 (23.5) | 0.28 |
| Air crescent | 2 (11.8) | 2 (11.8) | >0.99 |
| Airway-invasive form | 11 (64.7) | 7 (41.2) | 0.30 |
| 1. **Symptom duration: > 28 days (N=11)** | | | |
| **Imaging** | **Aspergillosis**  (N=7) | **Mucormycosis**  (N=4) | **P-value** |
| Size of representative lesion ≥4 cm | 5 (71.4) | 3 (75.0) | >0.99 |
| Macronodule-multiple | 3 (42.9) | 2 (50.0) | >0.99 |
| Consolidation | 4 (57.1) | 4 (100.0) | 0.23 |
| Halo sign | 0 (0.0) | 2 (50.0) | 0.10 |
| Reverse halo sign | 0 (0.0) | 3 (75.0) | **0.02** |
| Cavity | 3 (42.9) | 0 (0.0) | 0.23 |
| Air crescent | 0 (0.0) | 0 (0.0) | >0.99 |
| Airway-invasive form | 3 (42.9) | 1 (25.0) | >0.99 |

**Supplemental Table 5** Comparison of computed tomography findings between pulmonary mucormycosis (PM) and invasive pulmonary aspergillosis (IPA), among patients who did not receive antifungal medication

| **Imaging** | **Aspergillosis**  (N=40) | **Mucormycosis**  (N=21) | **P-value** |
| --- | --- | --- | --- |
| Size of representative lesion ≥4 cm | 15 (37.5) | 12 (57.1) | 0.14 |
| Macronodule-multiple | 14 (35.0) | 8 (38.1) | 0.81 |
| Consolidation | 25 (62.5) | 18 (85.7) | 0.05 |
| Halo sign | 12 (30.0) | 12 (57.1) | **0.03** |
| Reverse halo sign | 4 (10.0) | 9 (42.9) | **<0.01** |
| Cavity | 7 (17.5) | 3 (14.3) | >0.99 |
| Air crescent | 1 (2.5) | 2 (9.5) | 0.27 |
| Airway-invasive form | 24 (60.0) | 5 (23.8) | **0.01** |

**Supplemental Table 6** Predictors of pulmonary mucormycosis, among patients who did not receive antifungal medication, by univariable and multivariable logistics regression analysis (model I)

| **Imaging** | **Univariable** |  | **Multivariable^1^** |  |
| --- | --- | --- | --- | --- |
|  | Odd ratio  (95% CI) | P-value | Odd ratio  (95% CI) | P-value |
| Size ≥4cm | 2.222 (0.758-6.514) | 0.146 |  |  |
| Micronodule-multiple | 1.143 (0.383-3.414) | 0.811 |  |  |
| Consolidation | 3.600 (0.906-14.306) | 0.069 |  |  |
| Halo | 3.111 (1.038-9.322) | **0.043** |  |  |
| RHS | 6.750 (1.755-25.956) | **0.005** | 6.750 (1.755-25.956) | **0.005** |
| Cavity | 0.786 (0.181-3.415) | 0.748 |  |  |
| Air crescent | 4.105 (0.350-48.161) | 0.261 |  |  |
| Airway invasive form | 0.208 (0.064-0.683) | **0.010** | 0.264 (0.076-0.916) | **0.036** |

^1^The forward selection method was used to build model I as a stepwise regression. A p-value of <0.05 was considered to indicate statistical significance.

**Supplemental Table 7** Predictors of pulmonary mucormycosis, using host factors, diagnostic testing and CT findings, by univariable and multivariable logistics regression analysis (model I)

| **Imaging** | **Univariable** |  | **Multivariable^1^** |  |
| --- | --- | --- | --- | --- |
|  | Odd ratio  (95% CI) | P-value | Odd ratio  (95% CI) | P-value |
| **Host factors** |  |  |  |  |
| Prolonged neutropenia^2^ | 1.685 (0.552-5.143) | 0.359 |  |  |
| Chronic lung diseases | 0.211 (0.072-0.619) | **0.005** | 0.183 (0.050-0.671) | **0.010** |
| Solid organ transplant | 0.321 (0.116-0.893) | **0.029** |  |  |
| Solid tumor | 0.623 (0.201-1.931) | 0.413 |  |  |
| Hematologic malignancy | 3.929 (1.612-9.576) | **0.003** |  |  |
| HSCT^3^ | 3.240 (1.040-10.096) | **0.043** |  |  |
| Diabetes mellitus | 1.997 (0.829-4.812) | 0.123 |  |  |
| Chronic kidney disease | 2.769 (0.870-8.810) | 0.085 |  |  |
| Autoimmune disease | 0.879 (0.077-10.064) | 0.917 |  |  |
| **Diagnostic testing** |  |  |  |  |
| GM positive by EORTC^4^ | 0.172 (0.059-0.505) | **0.001** | 0.079 (0.019-0.332) | **0.001** |
| Aspergillus on non-sterile culture | 0.088 (0.019-0.399) | **0.002** |  |  |
| **CT findings** |  |  |  |  |
| Size ≥4cm | 2.222 (0.758-6.514) | 0.146 |  |  |
| Micronodule-multiple | 1.143 (0.383-3.414) | 0.811 |  |  |
| Consolidation | 3.600 (0.906-14.306) | 0.069 |  |  |
| Halo | 3.111 (1.038-9.322) | **0.043** | 4.570 (1.277-16.356) | **.020** |
| RHS | 6.750 (1.755-25.956) | **0.005** | 5.023 (1.292-19.530) | **.020** |
| Cavity | 0.786 (0.181-3.415) | 0.748 |  |  |
| Air crescent | 4.105 (0.350-48.161) | 0.261 |  |  |
| Airway invasive form | 0.208 (0.064-0.683) | **0.010** |  |  |

^1^The forward selection method was used to build model I as a stepwise regression. A p-value of <0.05 was considered to indicate statistical significance.

^2^Prolonged neutropenia is defined by <0.5 × 10^9^ neutrophils/L [<500 neutrophils/mm^3^] for >10 days

^3^Hematopoietic stem cell transplant

^4^One of the following achieved to meet the EORTC (+) criteria: (1) single serum or plasma: ≥ 1.0; (2) BAL fluid: ≥1.0; (3) single serum or plasma: ≥0.7 and BAL fluid ≥0.8; (4) CSF: ≥1.0

**Supplemental Figure 1.**


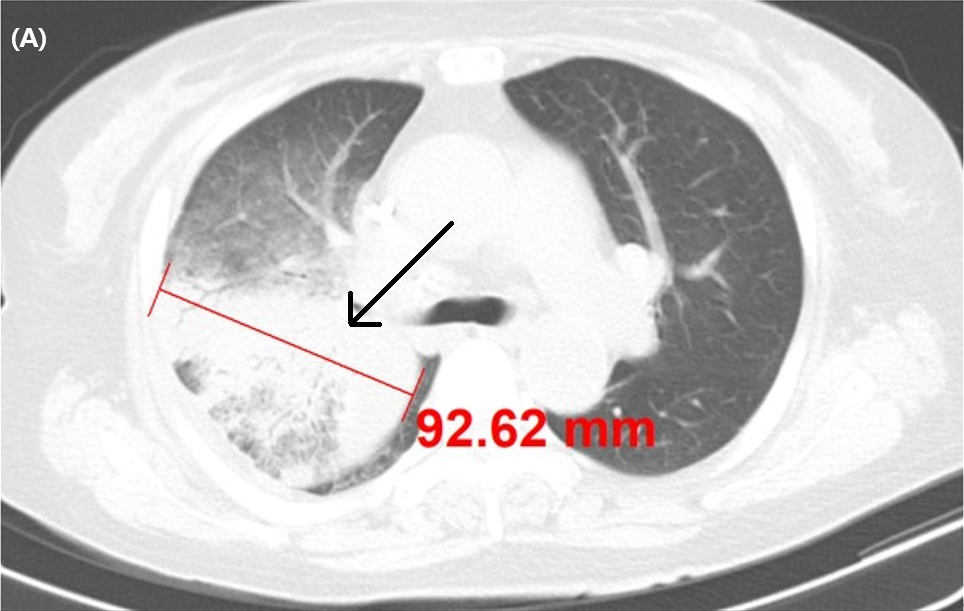


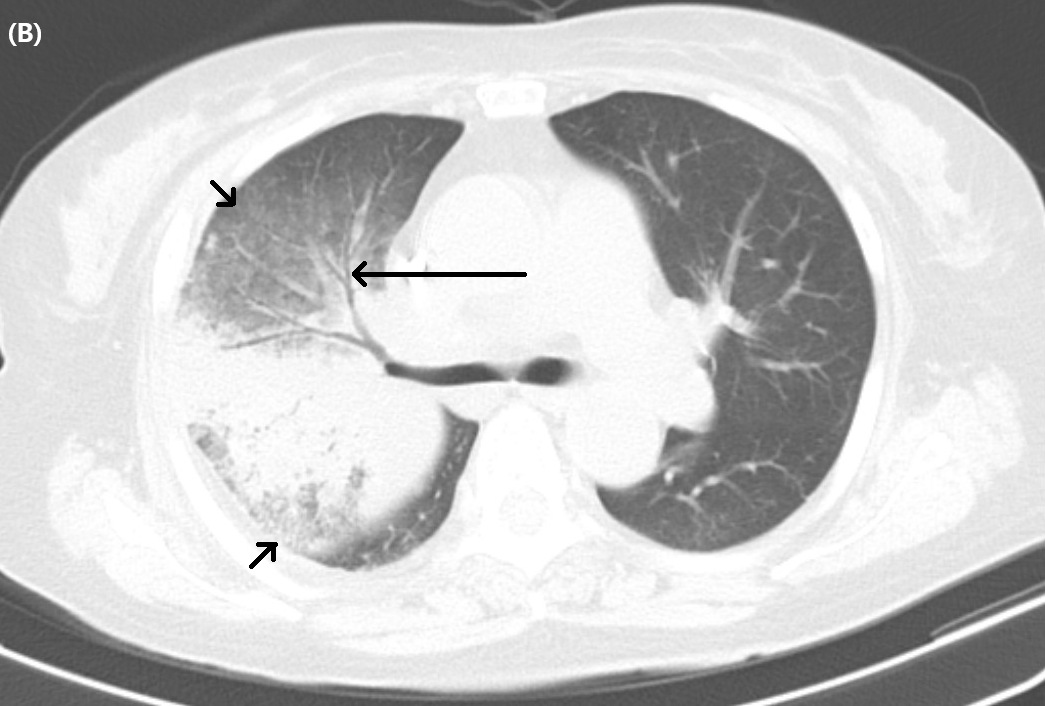


**Legend:** Conventional chest CT images obtained in a 63-year-old female with pulmonary mucormycosis who has history of acute myeloid leukemia.

(A) CT transverse image with lung setting (3-mm thickness) obtained at the level of carina shows a mass-like consolidation (arrow) with surrounding ground-glass opacity measuring 9.3 cm in the right upper lobe. The size of the representative lesion (target lesion) is the long diameter of the mass or consolidation (excluding ground-glass opacity).

(B) CT transverse image with lung setting (3-mm thickness) obtained at the level of both main stem bronchi. Note a consolidation with outer ground-glass opacity, representing ‘halo-sign’ (short arrows) and air bronchogram (long arrow) in the right upper lobe.

**Supplemental Figure 2.**


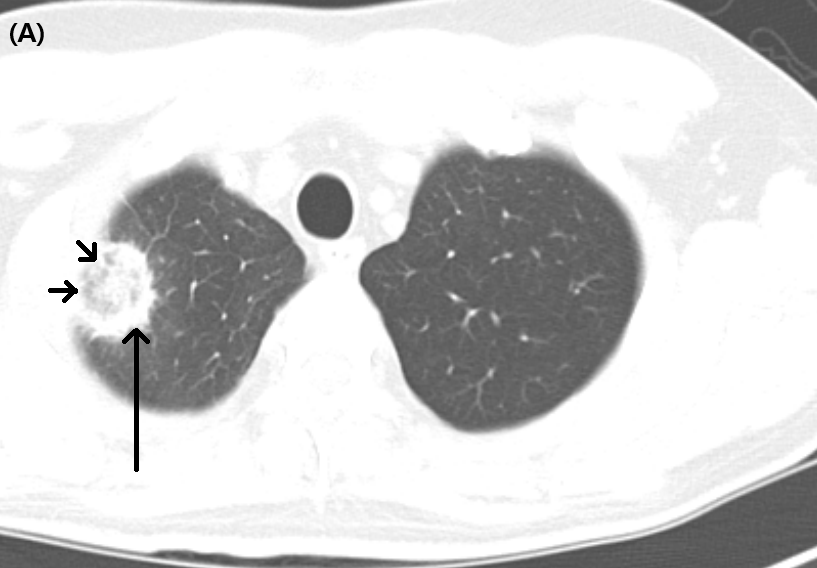


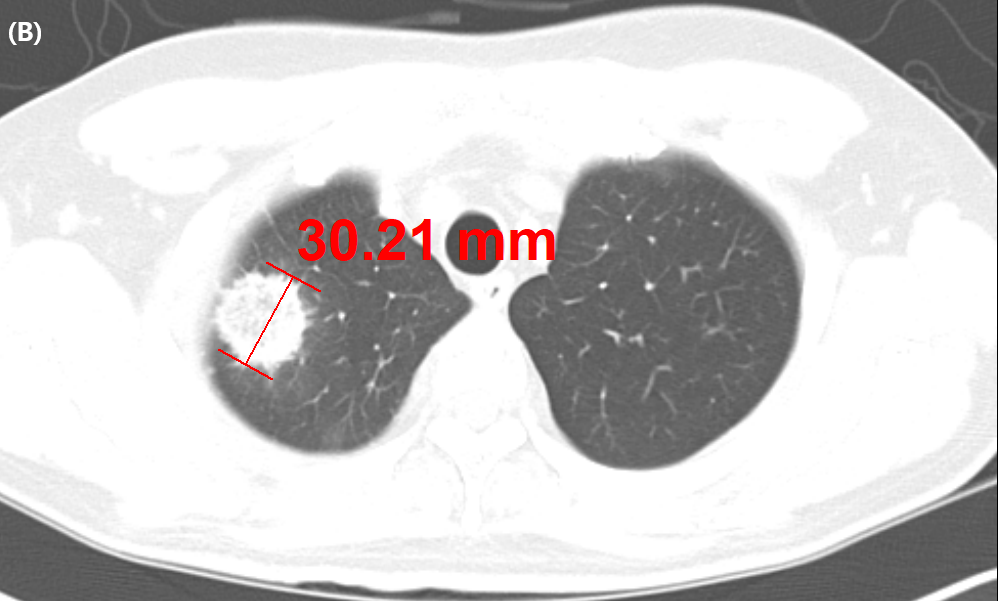


**Legend:** Conventional chest CT images obtained in a 38-year-old male with pulmonary mucormycosis who has history of acute myeloid leukemia.

(A) CT transverse image with lung setting (3-mm thickness) obtained at the level of aortic arch vessels shows a mass-like consolidation with internal low attenuating portion and intermingled ground-glass opacity (short arrows) representing ‘reverse halo sign’ (long arrow), located in the right upper lobe.

(B) CT transverse image with lung setting (3-mm thickness) obtained at the level of both brachiocephalic veins. Note a 3.0-cm spiculated margined mass, located peripherally in the right upper lobe (distribution of dominant area: peripheral). The size of representative lesion (target lesion) is the long diameter of the lesion (excluding ground-glass opacity).

**Supplemental Figure 3.**


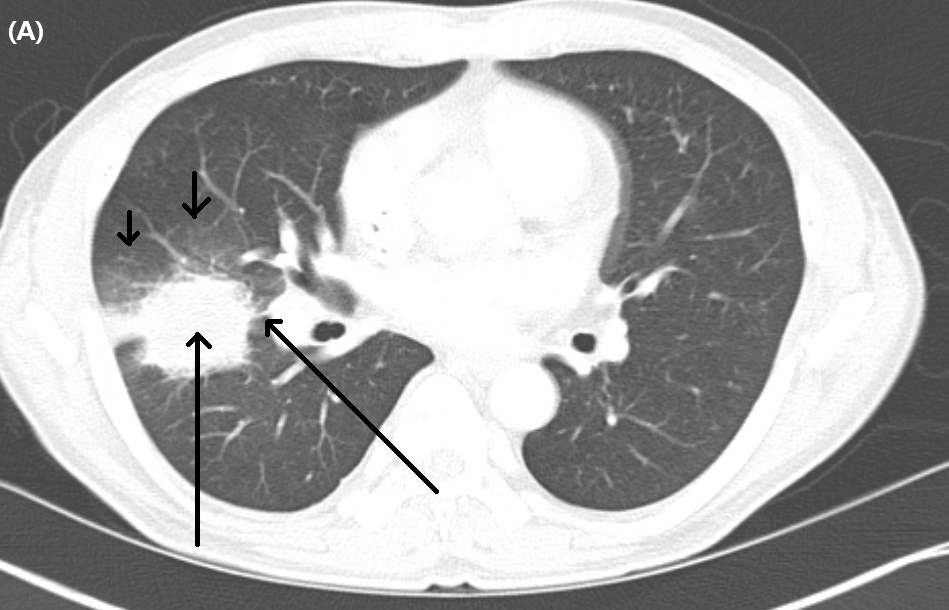


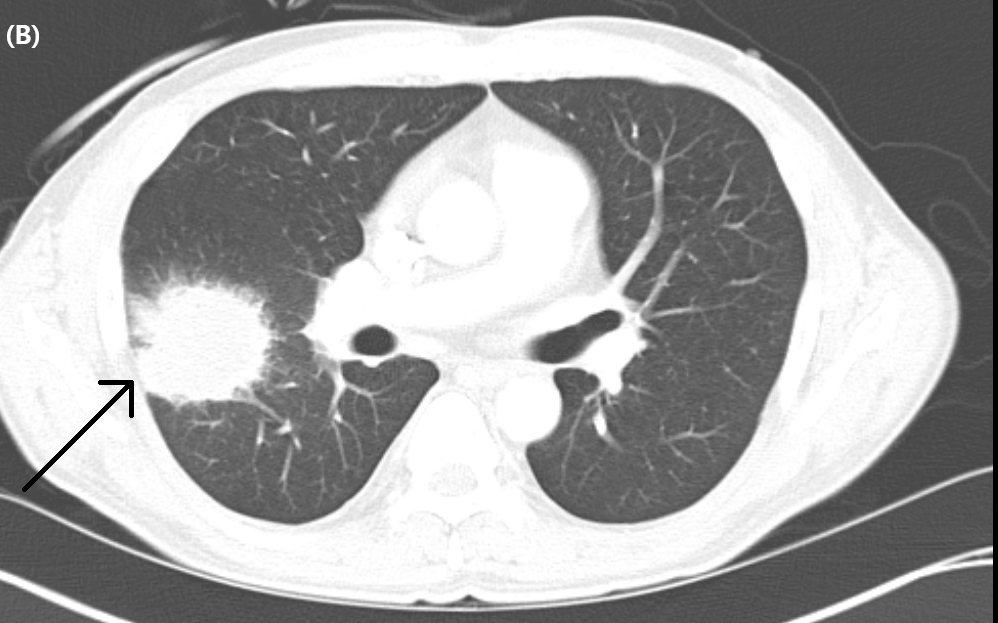


**Legend:** Conventional chest CT images obtained in a 45-year-old male with pulmonary mucormycosis who has a history of acute myeloid leukemia and had undergone allogenic hematopoietic stem cell transplant 4 months prior.

(A) CT transverse image with lung setting (3-mm thickness) obtained at the level of right middle lobar bronchus shows a lobulated mass-like consolidation with surrounding ground-glass opacity (short arrows) in right middle lobe. The representative lesion traverse to the superior segment of right lower lobe through major fissure (long arrows).

(B) CT transverse image with lung setting (3-mm thickness) obtained at the level of the right lower lobar bronchus. Note the tissue plane invasion where the same representative lesion abutted extra-pleural fat of the chest wall (arrow).

**Supplemental Figure 4.**


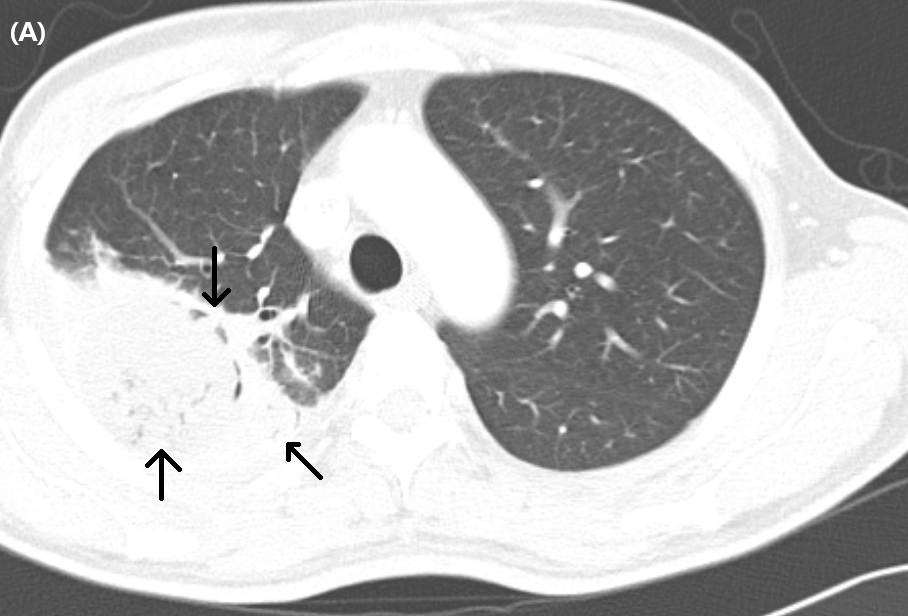


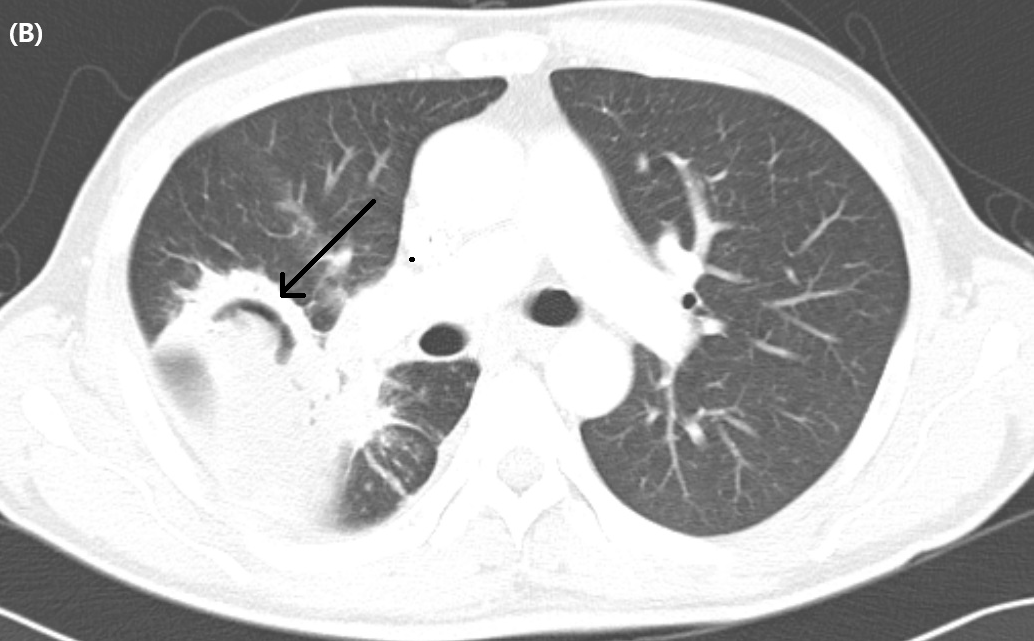


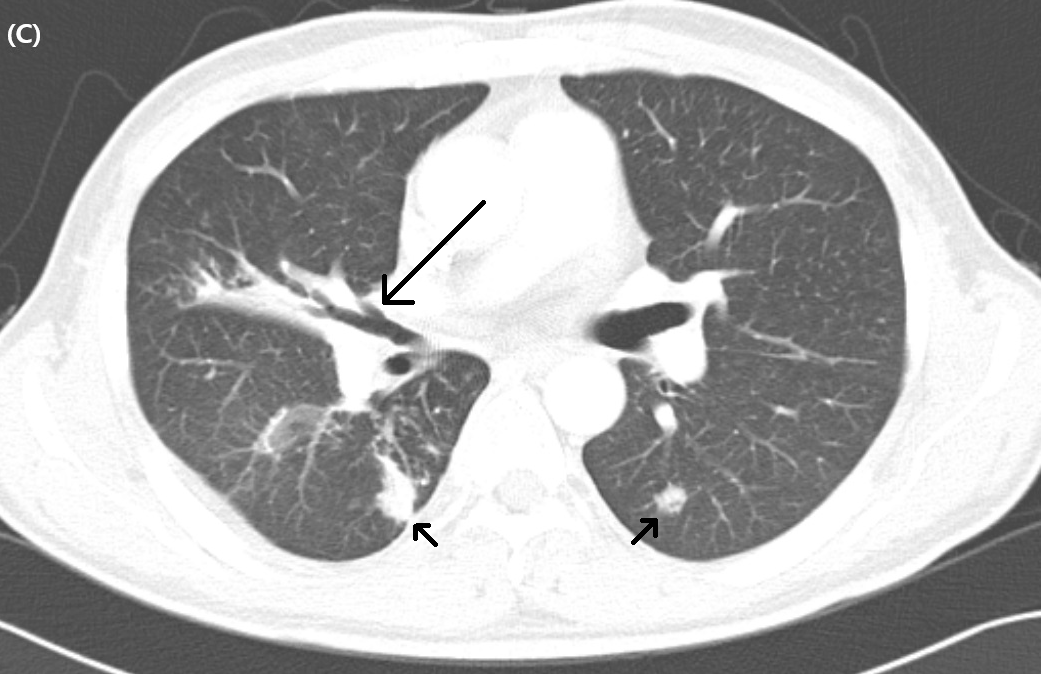


**Legend:** Conventional chest CT images obtained in a 43-year-old male with invasive pulmonary aspergillosis who has history of chronic myeloid leukemia, which progressed into acute lymphoblastic leukemia.

(A) CT transverse image with lung setting (3-mm thickness) obtained at the level of aortic arch shows an outer mass like consolidation with internal heterogeneous necrotic low attenuation (arrows).

(B) CT transverse image with lung setting (3-mm thickness) obtained at the level of the pulmonary trunk. Note the ‘air crescent sign’ (arrow) in the cavitary lesion.

(C) CT transverse image with lung setting (3-mm thickness) obtained at the level of the right middle lobar bronchus shows tracheobronchial wall thickening (long arrow) and multiple broncho-centric nodules with segmental distribution (short arrows)

**Supplemental Figure 5.** The receiver operating characteristic (ROC) curve for the cutoff of the representative lesion size


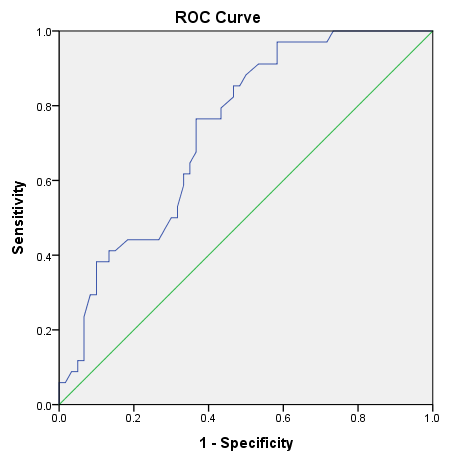


AUC area 0.735; optimal cut-off: 3.85 cm; Sensitivity: 76.5%; Specificity: 63.3%; The cutoff for the size of representative lesion was chosen as 4 cm for logistics regression analysis, which was rounded to the nearest integer from 3.85 cm.

**Supplemental Figure 6.** The receiver operating characteristic (ROC) curve for the cutoff of the prognostic point scoring system of pulmonary mucormycosis


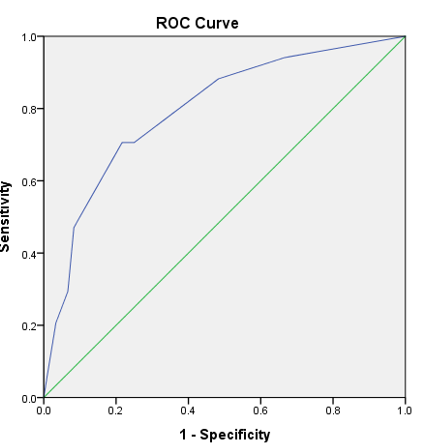


AUC (area under curve) 0.80 (95% CI 0.75–0.85); the optimal cut-off: score 8 (sensitivity 71%, specificity 78%).
